# Education, leadership and partnerships: nursing potential for Universal Health Coverage

**DOI:** 10.1590/1518-8345.1092.2673

**Published:** 2016-03-04

**Authors:** Isabel Amélia Costa Mendes, Carla Aparecida Arena Ventura, Maria Auxiliadora Trevizan, Leila Maria Marchi-Alves, Valtuir Duarte de Souza-Junior

**Affiliations:** 1 PhD, Full Professor, Escola de Enfermagem de Ribeirão Preto, Universidade de São Paulo, PAHO/WHO Collaborating Centre for Nursing Research Development, Ribeirão Preto, SP, Brazil; 2 PhD, Associate Professor, Escola de Enfermagem de Ribeirão Preto, Universidade de São Paulo, PAHO/WHO Collaborating Centre for Nursing Research Development, Ribeirão Preto, SP, Brazil; 3 PhD, Professor, Escola de Enfermagem de Ribeirão Preto, Universidade de São Paulo, PAHO/WHO Collaborating Centre for Nursing Research Development, Ribeirão Preto, SP, Brazil; 4 Doctoral student, Escola de Enfermagem de Ribeirão Preto, Universidade de São Paulo, PAHO/WHO Collaborating Centre for Nursing Research Development, Ribeirão Preto, SP, Brazil

**Keywords:** Enfermería, Cobertura Universal, Educación, Liderazgo, Conducta Cooperativa, Salud Global

## Abstract

**Objective:**

to discuss possibilities of nursing contribution for universal health coverage.

**Method:**

a qualitative study, performed by means of document analysis of the World Health Organization publications highlighting Nursing and Midwifery within universal health coverage.

**Results:**

documents published by nursing and midwifery leaders point to the need for coordinated and integrated actions in education, leadership and partnership development.

**Final Considerations:**

this article represents a call for nurses, in order to foster reflection and understanding of the relevance of their work on the consolidation of the principles of universal health coverage.

## Introduction

Some countries consider health care to be a fundamental right, or a commodity. For over a century, universal health coverage has represented a dream come true in most developed countries, although it is still a goal to be achieved in developing countries^(^ 1 ^)^.

The consolidation of universal health coverage is directly related to multiple, complex factors internal and external to the health system, including economic, social, political, ethical and legal aspects. In this scenario, Article 25 of the Universal Declaration of Human Rights of 1948 states that "everyone has the right to a standard of living that is adequate for the health and well-being of himself and of his family, including food, clothing, housing and medical care and necessary social services, and the right to security in the event of unemployment, sickness, disability, widowhood, old age or other lack of livelihood in circumstances beyond his control"^(^ 2 ^)^. According to Carissa Etienne, Director of the Pan American Health Organization (PAHO), governments have a moral imperative to seek ways to improve equity and promote health and development, and universal coverage is the way to reach that^(^ 3 ^)^.

In this context, there are two clear reasons for a commitment to universal health care coverage: the first is linked to the right of each individual to health and health care, and the second refers to reflections of individual health problems for the community, and of the developing countries for the developed countries. Overall, the global society therefore has an interest in improving access to universal coverage in developing countries. In practice, despite government commitments, effective access to health care depends heavily on both economic and social conditions of the countries involved^(^ 4 ^)^.

Thus, universal health coverage is defined by access to the promotion of key interventions, prevention, treatment and rehabilitation for all, at an affordable cost, in the pursuit of achieving equity in access^(^ 5 ^)^.

Therefore, the goal of universal health coverage is to ensure that all people have the health care they need, without financial restrictions. This definition is aligned with the values and principles established by the concepts of Health for All and Primary Health Care^(^ 1 ^)^. Universal Health Coverage is based on the foundations agreed in the Constitution of the World Health Organization, in 1948, which declared health as a fundamental right of every human being, as well as the agenda established in Alma Ata in 1978.

Universal Health Coverage has a direct impact on people's health because access to services is a crucial component for sustainable development and poverty reduction, and a key element for reducing social inequities. It must therefore integrate the country's commitment to improving the well-being of its citizens^(^ 5 ^)^.

The following four publications resulted from discussions in different global forums: 2010 WHO report Health Systems Financing: the Path to Universal Coverage^(^ 6 ^)^; Bangkok's Statement on Universal Health Coverage, in January 2012^(^ 7 ^)^; Mexico City Political Declaration on Universal Health Coverage, adopted in April 2012^(^ 8 ^)^ and the Tunis Declaration on Money Value, Sustainability and Accountability in the Health Sector, approved in July 2012^(^ 9 ^)^. They culminated in the approval by the UN of Universal Health Coverage on December 12 2012, thereby recognizing health's role in achieving the international development goals and urging countries, civil society and international organizations to include universal health coverage in the global development agenda. The resolution reaffirmed WHO's leadership in supporting countries to respond to the challenges of the process of implementing universal coverage, considering health as a precondition, result, and indicator of three dimensions of sustainable development.

According to Margaret Chan, Director-General of WHO^(^ 10 ^)^, after the release of the 2010 Report (WHO), more than 60 developing countries requested consultation from the World Health Organization (WHO) for the implementation of universal coverage in their health systems. 

Experts suggest that a minimum package of basic interventions for universal health care coverage is defined, prioritizing concrete low-cost actions to deal with specific health problems in each location, according to their specificities^(^ 4 ^)^.

Also, in the discussions on the Millennium Development Goals (MDGs), advocates of universal health coverage envisioned the opportunity to incorporate their views on more solid and equitable health systems in the context of the post-2015 development agenda. Thus, the proposal of the Open-Ended Working Group on Sustainable Development Goals (SDGs) included universal coverage in its pre-MDGs project^(^ 11 ^)^.

Some factors must necessarily be considered by countries to achieve the universal health coverage target, including^(^ 5 ^)^:

- Efficient health care system that reaches the priority health needs by means of an integrated and focused attention on the people in order to: encourage people to become healthy and prevent diseases by facilitating access to health information; diagnose health conditions early, possessing the ability to treat diseases and help people in rehabilitation;
- Affordability and access to medicines and technologies for treating health problems;
- Recognition of the interdependence of health with other social determinants;
- Trained and motivated human resources to provide services that meet patients' needs based on the best evidence.

From this perspective, universal health coverage is not *per se* a guarantee of efficiency and effectiveness of care. In addition to political will, universal health coverage requires motivated people who have adequate resources for prevention, diagnosis, treatment and professional development, promoting the consolidation of a good governance culture, reflected in the health professional's posture and attitude^(^ 12 ^)^.

Among health professionals, nurses act as individuals, members, and coordinators of interprofessional teams, and are characterized by care centered on people that are closest to the communities where it is most required, participating in the improvement of health outcomes and cost-effective services^(^ 13 ^)^.

Nursing has features that potentiate its contribution for strengthening the quality of health systems, playing a key role in recognizing the importance of universal health coverage and its respective implementation, considering the different realities and national needs.

From this perspective, this article discusses possible nursing contributions to universal health coverage.

## Method

This was a qualitative study using document analysis of WHO publications that highlight Nursing and Midwifery in Universal Health Coverage. Three documents were used: Nursing and Midwifery Services Strategic Directions - SDNM^(^ 13 ^)^, WHO Global Forum for Government Chief Nursing and Midwifery Officers - WGFGCNO declaration^(14)^ and TRIAD Communiqué declaration^(^ 15 ^)^. Data were collected using a structured questionnaire applied to each document.

Data were analyzed using deductive content analysis^(^ 16 ^)^, which is a method of systematic research, whose main objective is the analysis of documents. This analysis favors the construction of knowledge, the adoption of new perspectives, and representation of facts^(^ 17 ^)^.

Each researcher analyzed data individually and, subsequently, the results independently obtained by each investigator was juxtaposed and discussed until consensus on emerging issues was reached. It was defined that the interpretation process and data discussion would be based on WHO official documents related to universal health coverage.

## Results

The document, Nursing and Midwifery Services Strategic Directions (2011 to 2015) - NMSSD, published in 2010, was developed based on research results led by the WHO, with the Global Advisory Group on Nursing and Midwifery (GAGNM), Global Network of WHO Collaborating Centers for Nursing and Midwifery (GNWHOCC), International Confederation of Midwives (ICM), International Council of Nurses (ICN), International Labor Organization (ILO) and Sigma Theta Tau International Honor Society for Nursing participation. The NMSSD is based on principles of: a)*ethical action*: planning and providing health-care services based on equity, integrity, fairness and respect for gender and human rights; b)*relevance*: developing health services and systems guided by health needs, evidence and strategic priorities; c) *ownership*: adopting a flexible approach to be implemented with local involvement that is designed to guide action at both the global and national levels; and d)*partnership*: working together on common objectives, acting collaboratively and supporting each other's efforts^(^ 13 ^)^.

The document established five key areas (KRA) of nursing practice:

KRA1: Strengthening of health systems and services

KRA 2: Nursing and midwifery policy and practice

KRA 3: Education, training and career development 

KRA 4: Nursing and midwifery workforce management

KRA 5: Partnership for nursing and midwifery services

KRA 1 defines the nursing contribution to the performance of health systems, focused on universal coverage, interfering in health outcomes by means of active engagement and leadership in the different decision-making levels for the establishment of related policies. It is subdivided into two objectives: 1.1. To give nurses and midwives a greater role in ensuring that the design, delivery and performance of health systems tally with the needs of the people and the social determinants of health; 1.2. To empower nurses and midwives to provide leadership at every level of the health system.

KRA 2 indirectly approaches universal health coverage because it focuses on nursing leadership and its relationship with different stakeholders, such as civil society, government, professional organizations, service providers and education, targeting its role in filling gaps in policy development. Considering universal health coverage as a gap in the health policies of countries, the area of nursing practice, which relates to educational programs, professional regulation, and research development, is relevant. The objectives of this KRA are: 2.1. To ensure that nursing and midwifery policies are an integral part of overall health policy-making; 2.2. To enhance the professional standing of nursing and midwifery; 2.3. To build up the evidence base for nursing and midwifery practice through research, and to make sure it is used when changing practice.

KRA 3 indirectly emphasizes universal health care coverage by means of development of institutional training capacity and nursing education, with the purpose of mobilizing and optimizing human, material, and financial resources. Its objectives are: 3.1. To ensure that pre-service and continuing education programs at every level of nursing and midwifery produce an adequate supply of competent practitioners to meet the country's need; 3.2. To ensure that nursing and midwifery education/training programs are equipped with adequate teaching resources; 3.3. To develop nursing and midwifery expertise through post-basic education, mentoring and other career development activities.

KRA 4 highlights the management of the nursing workforce in order to create an environment that promotes the achievement of health goals, public expectations, and scientific evidence. The objectives established for achieving this KRA 4 were: 4.1. To ensure that national development plans include appropriate health human resource strategies and promote equitable access to nursing and midwifery services; 4.2. to foster a positive work environment, with supportive supervision, for optimal nursing and midwifery workforce performance.

Finally, KRA 5 values the partnerships among organizations and formal and informal networks for the development of efficient and effective health systems. It has the following objectives: 5.1. To help governments support the strengthening of health systems through the development of sound stewardship and governance, especially in nursing and midwifery services; 5.2. To encourage stakeholders to participate in the implementation and monitoring of the strategic directions for strengthening nursing and midwifery services with a view to the strengthening of nursing and midwifery services through resource mobilization, awareness-raising and advocacy on priority issues; 5.3. To improve nursing and midwifery services through effective networking and partnerships with organizations and practice communities, making use of new technologies and other mechanisms.

The Declaration approved during the WHO Global Forum for Governmental Chief Nursing and Midwifery Officers - WGFGCNO^(^ 14 ^)^, on May 15 2014, on Nursing and Midwifery and the Universal Health Coverage establishes the strategies of these actors in their national contexts:

1. Leadership and Management;
2. Education and Training;
3. Collaborative Patternships.

The first strategy is subdivided into four actions: 1.1. Build political support at the highest level of the health system to ensure continuity in the pursuit of Universal Health Coverage; 1.2. Formulate nursing and midwifery policies that encapsulate the vision for UHC to ensure integrated people-centered services. 1.3. Develop and/or strengthen policies for improving the quality of education and training, recruitment, retention and deployment. 1.4. Develop evidence-based policies for effective and efficient nursing and midwifery workforce management.

To achieve the second strategy, the following actions are recommended: 2.1. Support educational institutions in developing and implementing curricula that take into account the quantity, quality and relevance of the nursing and midwifery workforce to meet the local and national changing health needs. 2.2. Build and sustain the technical capacity to ensure quality education and practice through continuing professional development programs. 2.3. Work and support partners' efforts to assess the gap between the need for a health workforce, actual supply, geographical distribution (supplies, skills mix and competencies) and the population's demand for health services.

Strategy three consists of the following actions: 3.1. Identify key partners including service users to support and build the capacity of the nursing and midwifery workforce to contribute to Universal Health Coverage; 3.2. Develop and support nursing and midwifery interventions that lead to improved access to health care services; 3.3. Develop strategies that support the creation of links between public, non-governmental and private sectors to minimize barriers of access to health services in rural and remote or hard to reach areas. 

The TRIAD Communiqué Declaration was approved in the end of the meeting organized by the triad of the WHO, ICN, and ICM, aiming to strengthen the nursing and midwifery workforce to support universal health coverage^(^ 15 ^)^. This document was based on the Resolutions of the World Health Assembly and on the WHO Report entitled, "The universal truth: no health without a workforce". It pointed out, as guidelines:

- Leadership and Policy Direction for Universal Health Coverage;
- Quantity, Quality, and Relevance of the Nursing and Midwifery Workforce; and
- Collaborative Patternships in the current economic realities and beyond.

By addressing Nursing Leadership, the Declaration reinforces the responsibility of professionals in improving the health of people and the indispensability of their involvement in political dialogue and decision-making on planning, development, and evaluation of services and policies. Regarding Quantity, Quality, and Relevance of the Workforce, the Declaration pointed out the need to increase the number of these professionals associated with quality education as essential factors to ensure universal health coverage. In regard to collaborative partnerships, the Declaration reaffirmed the importance of both mutual collaboration and financial and human resources sharing in the development of innovative approaches to handle the challenges and implement transformative actions that improve the security, quality and productivity of the provided service in according to the goal of universal health coverage.

**Figure 1:**
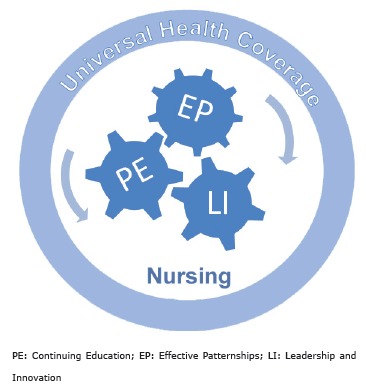
- Nursing Contribution to the Universal Health Coverage

From the analysis of the three documents developed by the Nursing and Midwifery leaderships within the WHO, ICN, and ICM, three themes are derived related to nursing's contribution to universal health coverage: *Continuing Education, Effective Partnerships and Leadership and Innovation*. (Figure 1 ).

## Discussion

### Nursing and its contribution to Universal Health Coverage

The three emerging themes emphasize the nursing contribution as a means to achieve the Universal Health Coverage aim, considering its main pillars: access to health care, coverage, health system gateway, rights-based approach, and protection from economic and social risk^(^ 18 ^)^.

The theme *Continuing Education* [KRA 3 SDNM, Strategy 2 WGFGCNO and Guideline Quantity, Quality and Relevance of Workforce Nursing and Obstetrics of TRIAD Communiqué] encompasses the basic and post-basic education, by means of curriculum with central contents, coordinated with health policies and local realities, in order to guarantee the minimum number of nurses in the health services recommended by the WHO, capable of qualified professional performance and sustained education programs throughout one's working life. Nurses' education is especially important, considering the global lack of these professionals, reinforced by the increasing service demand and associated with the rise in migration ^(^ 19 ^)^.

In 2001, the World Health Assembly approved Resolution 54.12, validating the WHO commitment and its member countries` with the expansion and strengthening of training of nurses and midwives. It is important to highlight that the Nursing and Midwifery Services Strategic Directions document (2002-2008) served as a guide for the implementation this national government's resolution. By meeting the needs expressed by the Member States, Resolution WHA 59.27 urges governments to implement programs to strengthen nursing and midwifery development, supporting both recruitment and retention, in addition to the active involvement of nurses and midwives in the health system development^(^ 20 ^)^. Also, as a result of this WHO movement, in 2009 the document "Global Standards for the Initial Education of Professional Nurses and Midwives" was created^(^ 21 ^)^.

Considering the diversity of nursing education programs in the world, the WHO has proposed the adoption of global standards by establishing evidenced-based criteria and expertise for nursing professional training that provides skilled care and promotes relevant health outcomes for the attended population^(^ 21 ^)^.

It is also important to highlight that the nursing professional education must consider the wider health context, including its social determinants, and the principles of sustainable development. In this backdrop, the structural iniquities also establish differences in the local health priorities, requiring the development of both competencies and specifics skills in the nursing health professional's education^(^ 22 ^)^.

From this perspective, the Action Plan, established by the WHO for the health system governance for universal coverage, values human resource development as a fundamental condition of the system's effectiveness^(^ 23 ^)^. Therefore, the investment in education over the life of these valuable human resources is vital, in order to offer quality health services to the users: the possible health outcomes depend on its valuation. And here, Nursing deserves emphasis and special attention from governments, managers, education and health leaders: it is a profession that is considered to be the backbone of the health system, not only because of its majority representation in the health workforce, but also because of its presence, performance and permanence (24 hours) in services, coordinating care and representing the link between the members of the health team^(^ 24 ^)^.

The theme *Leadership and Innovation* [KRA 1, KRA 2 e KRA 4 SDNM, Strategy 1 WGFGCNO, Directive Leadership and Policy Direction for the Universal Health Coverage of the TRIAD communiqué] points to the exercise of leadership and innovative performance of nurse, focused on his/her participation in the development of health policy, management of health services, valuation of its human resources, and a favorable environment in which to work. 

The competencies in nursing leadership must be cultivated for leaders to develop skills to ensure nursing's contribution to universal health coverage. It is therefore important to ensure nursing participation in the establishment of policies, strategies and clear goals for access to health coverage, as advocates of individual and social rights of the population, aimed at protecting economic and social risks. The technical expertise of the nurse, the size of its contingent and especially its proximity to the health services users, daily experiencing their needs, strengths and weaknesses, legitimize the imperative of this participation.

The World Health Organization recognizes the need for clearer examples of Nursing and Midwifery leadership in the health ministries. It is recommended that in the near future they act in teams and lead nursing and midwifery processes, highlighting that the approach of talent management is adopted to put them in strategic leadership^(^ 25 ^-^ 26 ^)^.

Leadership is exercised by means of support systems that promote that nurses are allowed to perform to their full ability, their contribution for improving the health outcomes, their participation in their own professional development, their satisfaction and recognition for the work they perform^(^ 19 ^)^. Nursing leadership is also expressed by means of innovating based on research results, and the entrepreneurial activities towards services` resolubility. 

Leaders are important, but leadership is even more so: a single leader can make a difference and produce better than expected results, but the collective leadership brings together leaders at all organizational levels through shared and sustainable actions. With the development of leaders, their organizations also progress, becoming more capable of sustaining the changes required by them. This is a permanent commitment with personal change and the leadership cultivation culture affecting all organizational leaders, therefore, leadership sustainability^(^ 27 ^)^.

 The nurse who is committed to current policies seeks constant self-improvement and the involvement of other leaders, being recognized as a leader in the healthcare system, assuming responsibility for sustainable leadership. Good leaders are believed to become even better when they are aware and convinced of the changes they know they need to make. Thereby, nurses need to commit to the implementation of programs that contribute to the consolidation of universal health coverage.

Data from the WHO demonstrate that, despite the process already achieved, health service coverage and the protection against financial risks are still far short of the goal of universal coverage, due to several factors, including difficulties understanding the association between the service coverage and health. These gaps can be filled by means of development of research on the topic^(^ 28 ^)^. It is recommended, therefore, that nurses develop research focusing on improving health service coverage, protection against financial risks and in outlining of indicators for data generation to monitor the progress in the universal coverage policy. In sum, research should focus on the nursing actions for achieving universal health coverage and the effects of these interventions.

Universal health coverage represents a means for improving the health conditions of people and the promotion of their development. Studies performed on the volu can play a key role in nursing actions implemented in the context of the MDGs, as well as being a support for discussion of the post-2015 sustainable development agenda^(^ 28 ^)^.

The subject, *Effective Partnerships* [KRA 5 SDNM, Strategy 3 WGFGCNO and Guidelines Collaborative Partnerships in the current economic and social situation and TRIAD Communiqué], reinforces the importance of nursing collaboration with different stakeholders, including the government, civil society, and professional organizations.

In a context of coping with difficulties with the consolidation of universal health coverage by various countries, development of partnerships emerges as a viable alternative to achieving policy directives focusing on social welfare, by means of joint work which is impossible to be performed by organizations separately^(^ 29 ^)^. The seed of necessity and the potential emergence of interdependence relationships, generating effective actions to narrow the relationship of the actors involved in care for system users, are identified in the scope of health organizations. Nurses, as links between the system services, exercise leadership which values diversity and strengthening the human power of all members, including the user^(^ 30 ^)^. Teamwork is recognized as central to the concept of partnership, both in the workplace and in the inner workings of both professions^(^ 31 ^)^.

Considering the relevance of collaborative work, in 2010, the WHO published a document highlighting the need to stimulate interprofessional collaboration in education as an innovative strategy to deal with the global crisis of the health workforce^(^ 32 ^)^. Therefore, collaborative practice in health care was defined as an integration of different professionals in the work with patients, their families, caregivers, and communities, aiming to offer high-quality care. The same concept can be adopted in the idealization and implementation of the partnerships for universal health coverage.

This concept can be operationalized, to the extent that vocational education systems for health are integrated and based on the same principles, encouraging joint learning based in collaboration.

Nursing uniquely performs within the collaboration context, with a subsystem of the health workforce sharing values centered especially on the care of people, complementing and supporting other health professionals.

The partnerships with an effective participation of nurses enable the successful implementation of the Universal Health Coverage, by means of interventions that ensure the promotion and restoration of health conditions of people, in order to value life and human dignity.

## Final Considerations

Universal health coverage is the greatest challenge currently faced by health systems in developed and developing countries. In a context characterized by growing discussions on possible ways to achieve this, in the political, economic, legal and social sectors, this paper contributes with a discussion of the potential for nurses' action aimed at the achievement of universal health coverage. Nursing will be more valuable if it can demonstrate, by means of research, the effects of its interventions for achieving universal health coverage.

The documents published by the leaders in nursing and midwifery indicate the need for coordinated and integrated actions of education, leadership and partnership development. Therefore, this article represents a call for nursing, in order to foster reflection and understanding of the relevance of its work, for the consolidation of the principles of universal health coverage.
